# Orthographic learning in children with isolated and combined reading and spelling deficits

**DOI:** 10.1080/09297049.2018.1470611

**Published:** 2018-05-07

**Authors:** Heike Mehlhase, Sarolta Bakos, Karin Landerl, Gerd Schulte-Körne, Kristina Moll

**Affiliations:** a Department of Child and Adolescent Psychiatry and Psychotherapy, University Hospital Munich, Munich, Germany; b Institute of Psychology, University of Graz, Graz, Austria

**Keywords:** dyslexia, orthographic learning, reading deficit, spelling deficit, children

## Abstract

Dissociations between reading and spelling problems are likely to be associated with different underlying cognitive deficits, and with different deficits in orthographic learning. In order to understand these differences, the current study examined orthographic learning using a printed-word learning paradigm. Children (4th grade) with isolated reading, isolated spelling and combined reading and spelling problems were compared to children with age appropriate reading and spelling skills on their performance during learning novel words and symbols (non-verbal control condition), and during immediate and delayed reading and spelling recall tasks. No group differences occurred in the non-verbal control condition. In the verbal condition, initial learning was intact in all groups, but differences occurred during recall tasks. Children with reading fluency deficits showed slower reading times, while children with spelling deficits were less accurate, both in reading and spelling recall. Children with isolated spelling problems showed no difficulties in immediate spelling recall, but had problems in remembering the spellings 2 hours later. The results suggest that different orthographic learning deficits underlie reading fluency and spelling problems: Children with isolated reading fluency deficits have no difficulties in building-up orthographic representations, but access to these representations is slowed down while children with isolated spelling deficits have problems in storing precise orthographic representations in long-term memory.

Word-specific orthographic representations are defined as “detailed associations between the spoken and the written form of words in long-term memory” (de Jong & Messbauer, 2011). According to Share’s self-teaching hypothesis (Share, 1995), these word-specific lexical representations are built-up during reading development by repeatedly applying letter-to-sound rules to translate letters or letter-groups (graphemes) into their corresponding sounds when decoding unfamiliar words. Thus, repeated decoding of initially unfamiliar words leads to the acquisition of word-specific orthographic knowledge, enabling orthographically correct spelling as well as accurate and fast word recognition by sight word reading (Ehri, 1992).

It has been suggested that individuals with reading disorder (i.e., dyslexia) show deficits in building-up these word-specific representations. Evidence for such deficits is based on studies analyzing orthographic learning in individuals with dyslexia compared to typically developing readers (Bailey, Manis, Pedersen, & Seidenberg, 2004; Castles & Holmes, 1996; Ehri & Saltmarsh, 1995; Wang, Marinus, Nickels, & Castles, 2014). Different learning tasks were used (mostly repeated reading of novel words) in order to assess the build-up of orthographic representations. Whether children have succeeded in acquiring orthographic representations has been examined in different tasks: reading performance, spelling performance (reproduction of the target spelling), and orthographic choice task (selection of the target spelling among four alternatives). These studies showed that children with dyslexia need more trials to learn to read novel words as compared to typical readers (Ehri & Saltmarsh, 1995). During the recall phase dyslexic readers are less accurate when reading previously learned words (Ehri & Saltmarsh, 1995) and make more errors in spelling learned words (Bailey et al., 2004; Ehri & Saltmarsh, 1995; Wang et al., 2014) or recognizing the correct spelling during orthographic choice tasks (Castles & Holmes, 1996; Wang et al., 2014). Thus, findings indicate that individuals with dyslexia are impaired when learning the spelling of novel words and that impaired orthographic learning affects both reading and spelling skills.However, word-specific orthographic representations might not only be important for accurate reading and spelling but may also be relevant for increasing reading fluency, as words that have been stored in long-term memory no longer have to be decoded serially but can be accessed as a whole. Evidence for the role of orthographic processing for fluent reading comes from consistent orthographies (Reitsma, 1983) where reading fluency is the main criterion for assessing reading skills as reading accuracy is close to ceiling after one year of reading instruction (Seymour, Aro, & Erskine, 2003). Reitsma (1983) compared orthographic learning in children by using a reading task in which target words were embedded in sentences and the number of target exposures was varied. Dyslexic children did not show an advantage (i.e. increase in reading speed) for previously learned words over pseudohomophonic spellings (graphemic alteration without altering the pronunciation) of these words compared to control children.

Thus, building-up word-specific orthographic representations seems to be important for reading accuracy, reading fluency, as well as for spelling and deficits in orthographic processing are likely to be associated with both, reading and spelling problems. This raises the question of how dissociations between reading and spelling deficits can be explained? In German, isolated deficits in reading (fluency) or spelling and at the same time age-appropriate performance in the unaffected literacy skill have been shown to be as frequent as combined reading and spelling problems with prevalence rates of 6% for isolated deficits in reading fluency, 7% for isolated deficits in spelling, and 8% for combined reading fluency and spelling deficits (cRSD; Landerl & Moll, 2010; Moll, Kunze, Neuhoff, Bruder, & Schulte-Körne, 2014; Moll & Landerl, 2009). Moreover, isolated deficits in reading fluency vs. spelling have been associated with different cognitive profiles (Moll & Landerl, 2009; Wimmer & Mayringer, 2002). While children with isolated reading fluency problems showed deficits in rapid automatized naming (RAN: serial naming of repeated items presented in lines or columns) but were unimpaired in phonological awareness (PA; e.g., phoneme deletion), children with spelling problems showed the opposite profile with deficits in PA (at least at the beginning of literacy instruction) but intact naming speed. Given, that PA has been shown to be a precursor for learning grapheme–phoneme correspondences (Frith, 1985; Perfetti, 1997) and for building-up word-specific orthographic representations (Share, 1995), the following assumptions can be made for the two isolated deficit groups: Children with isolated reading fluency deficits (iRD) seem to have word-specific representations available, as they show no difficulties in spelling and, in line with their cognitive profile, have no difficulties in PA. However, this does not mean that orthographic processing is unimpaired in children with iRD. Their deficits in reading fluency and naming speed may indicate that access from the visually presented word or item in the naming task (picture, color, letter, or digit) to the corresponding phonological representations may be disrupted or slowed down. Evidence for deficient access to otherwise intact representations of speech sounds has been provided by Boets et al. (2013). On the other hand, children with isolated spelling deficits (iSD) have problems in building-up and/or retaining word-specific orthographic representations, as they show problems in spelling and also have difficulties in PA at least at the beginning of literacy instruction. They presumably have a reduced orthographic lexicon and/or have degraded orthographic representations that might be sufficient for word recognition during reading but not for spelling. However, access to these representations seems to be intact, as they show no problems in reading fluency. Due to their relevance for fast reading, the reduced or degraded orthographic representations in children with iSD might lead to slightly reduced reading times in longer words.

In order to test these assumptions and to identify the exact nature of the orthographic processing deficit associated with reading vs. spelling problems, the current study examined how orthographic representations are acquired and what processes (learning, retaining, recognition and production) are affected in children with reading fluency vs. spelling problems. To this end we developed an orthographic learning task. Learning processes were investigated during a learning phase where children were asked to memorize the names and spellings of a set of alien creatures while performing an orthographic-visual paired associate learning task. In this task children had to map visual-orthographic information (spelling of the novel word) to visual-semantic information (picture of the alien). This paradigm was used in order to give children the opportunity to build-up a corresponding orthographic representation of the spelling by repeatedly seeing, reading and assigning the alien names. After this learning phase, a spelling task was given to investigate production of the learned spellings, and a reading task to examine word recognition. In order to investigate the retaining process, reading and spelling was re-tested again 2 hours after learning. For the orthographic learning task we used pseudowords that take into account the specific characteristics of German orthography, which is asymmetric and rather consistent in the reading direction, but less consistent in the spelling direction. German orthography consists of a high number of orthographic markers, which have to be memorized in order to spell a word correctly. Particularly inconsistent and complex is the orthographic marking of vowel length (Landerl & Reitsma, 2005). Consequently, the current study used pseudowords containing long vs. short vowels. By analyzing children’s misspellings, we hoped to gain information about how well children are able to differentiate between short- and long-vowel phonemes and how well they are able to apply the appropriate orthographic markers. As described above, word-specific representations may also be relevant for reading. Although, decoding words letter by letter might be sufficient to achieve high reading accuracy rates in the consistent German orthography, building-up word-specific representations may still be important for increasing reading speed as words no longer have to be decoded serially but can be accessed as a whole (Rau, Moeller, & Landerl, 2014).

Taken together, previous findings show impaired orthographic learning in children with dyslexia. However, the following aspects are still unresolved and are targeted in the current study:

First, previous studies did not differentiate between children with isolated reading fluency and spelling problems. Since these deficits occur as frequently as combined problems (at least in consistent orthographies like German), it is of great importance to understand these dissociations. The results can help to further specify theoretical models of written language processing and are relevant for intervention as findings can provide the basis for training programs that are specifically tailored toward individual deficit profiles. Thus, the current study included four groups: children with iRD, iSD, cRSD, and typically developing children (TD).

Second, the current study was set-up to differentiate between problems of forming and storing word-specific orthographic representations and is therefore divided into a learning phase (building-up word-specific representations), an immediate recall phase (short-term memory) and a delayed recall phase (storage in long-term memory 2 hours after learning).

Thirdly, the majority of studies focused on word learning only but did not assess non-verbal learning. In order to differentiate between general learning problems and learning problems that affect orthographic word learning only, the current study included a non-verbal control condition with symbols in addition to the word learning condition. Performance of symbol learning was also assessed during learning phase, immediate recall (short-term memory) and delayed recall (long-term memory).

Finally, we conducted an error analysis for misspelled words in order to look closer at the nature of spelling mistakes and to identify potential qualitative differences between the groups. Given that orthographic marking of vowel length is particularly complex in German orthography, the qualitative analysis focused on how well children are able to represent vowel duration in their spelling attempts.

The following research questions were addressed:

1. Are there group differences in learning during the learning phase? Deficits in the learning phase would reflect problems with mapping visual-orthographic to visual-semantic information. Studies investigating paired associate learning by mapping visual to visual information using abstract symbols found no deficits in children with dyslexia (Litt & Nation, 2014; Messbauer & de Jong, 2003).
2. The main research question was to examine whether we can identify group differences in orthographic learning measured by reading and spelling in the immediate and delayed recall phases. The hypotheses for the reading and spelling tasks in the two recall phases were as follows:
  (2a) Based on group assignments we expected generally poorer performance in the spelling task (i.e. delayed recall) in the two groups of poor spellers compared to the other two groups. However, our main interest was to test whether poor performance in spelling results from difficulties to build-up orthographic representations (immediate recall) which in turn affects delayed recall or from difficulties to store (delayed recall) the correct spellings of recently seen novel pseudowords. For children with iRD we assumed that they are able to build-up word-specific representations (given their age-adequate spelling skills) and thus do not have problems to produce target spellings in the immediate and delayed recall tasks.
  (2b) For the reading task (recognition of word-specific representations) we expected all children to show relatively high reading accuracy due to the high consistency of German letter-sound correspondences. However, if difficulties in reading accuracy would arise, we assumed that they should appear among poor spellers (iSD and cRSD) since they were expected to have problems in building-up precise representations of previously learned words. These unprecise representations are likely to affect reading of novel pseudowords which are similar to previously learned pseudowords but differ in vowel length from the learned pseudowords. Children with iRD are characterized by accurate but slow reading in consistent orthographies. Therefore, we do not expect them to show deficits in reading accuracy of previously learned words.
  (2c) Furthermore, we expected that dysfluent readers (iRD and cRSD) read the learned words more slowly than age-appropriate readers (based on the selection criteria). If reduced reading speed can mainly be explained by a deficit in accessing orthographic representations we expect that both groups of dysfluent readers perform equally poorly. For children with iSD the predictions are less clear. As argued before, word-specific orthographic representations might not only be important for accurate reading and spelling but could also be relevant for fluent reading. In this case we would expect that the iSD group shows slightly longer reading times than TD controls.
3. Finally, we wanted to address the question whether group differences in recalling spellings are specific for memorizing verbal material or whether the groups also differ in recalling symbols (non-verbal control condition). Deficits in recalling symbols would indicate a more general learning deficit that is not specific to learning orthographic patterns. So far, only few studies have included a non-verbal control condition (Messbauer & de Jong, 2003; Schulte-Körne, Deimel, Bartling, & Remschmidt, 2004). Based on these findings it is more likely that the groups differ in the verbal condition only.

## Method

### Participants

The study is part of a larger project investigating dissociations between reading and spelling disorders. Participants were selected based on standardized classroom tests of non-verbal IQ (CFT 20-R (Weiss, 2006)), reading fluency (SLS 2–9 (Wimmer & Mayringer, 2014)) and spelling (DRT-3 (Müller, 2003)) at the end of grade 3. In addition, reading scores were verified by an individually administered standardized 1-minute word and pseudoword reading fluency test (SLRT-II (Moll & Landerl, 2010) at the end of grade 3 and again one year later at the end of grade 4. Spelling was also repeatedly assessed using a standardized German spelling test (DERET 3–4+ (Stock & Schneider, 2008) at the end of grade 4. This age group was selected because from grade 3 on children are increasingly taught the orthographic spelling rules in school.

Inclusion criteria were non-verbal IQ ≥ 85, German as first language, normal or corrected-to-normal vision, absence of neurological deficits and a score below the clinical cut-off for AD(H)D as measured by a standardized parental questionnaire (DISYPS-II (Döpfner et al., 2008).

The study was approved by the institutional review board of the local ethics

committee and was performed in accordance with the latest version of the Declaration of Helsinki and in compliance with national legislation. Parents and children were informed in detail about the study and gave their written consent. Children received vouchers in return for their participation.

Four groups of children were included in the present study: Children with isolated reading fluency deficits and age-adequate spelling (iRD), children with isolated spelling deficits, and age-adequate reading (iSD), children with combined reading fluency and spelling deficits (cRSD) and children with age-appropriate reading and spelling (TD). In a first step, children were classified as reading and/or spelling impaired if they scored below the 20th percentile on at least one of the classroom measures administered at the end of grade 3 (reading: SLS 2–9; spelling: DRT-3). Children with reading and spelling performance between the 25th and 75th percentiles were included in the control group. In a second step, a deficit in reading and/or spelling was confirmed based on an individually administered reading fluency test (SLRT-II) at the same time point (end of grade 3) and by individually administered reading and spelling tests one year later (end of grade 4). To be included in the reading or spelling deficit group, performance had to be at or below the 20th percentile on at least two out of four reading subtests (word- or pseudoword reading administered in grades 3 and 4) and on at least one out of the two spelling tests (DRT-3 and DERET 3–4+ administered in grade 3 and 4). In addition, children’s overall performance (average score) on the individually administered reading fluency tests (SLRT-II) and spelling tests (DRT-3 and DERET 3–4+) had to be at or below the 25th percentile. Age-adequate development in reading and spelling was assigned by scores at or above the 30th percentile on at least two out of four reading subtests and at least one out of two spelling tests. In line with DSM-5 (American Psychiatric Association, 2014) diagnostic features, including several tests and two measurement time points ensured that deficits are persistent. Although the cutoff criterion for reading and spelling was rather lenient (≤20th percentile), 70 children out of 73 with literacy problems included in the current study fulfilled diagnostic criteria for either reading disorder or isolated spelling disorder (i.e., scoring at least one standard deviation below the population mean on a standardized reading fluency and/or spelling test together with converging evidence from school reports or academic history).^1^1Analyses without the three children who did not fulfill diagnostic criteria did not change the results. We therefore report the results for the whole sample. This selection procedure resulted in four clearly defined groups (see reading and spelling average scores in Table 1). The final overall sample included 108 children: 17 children with iRD, 21 with iSD, 35 with cRSD and 35 typically developing controls (TD).

**Table 1.** Descriptive statistics (means and standard deviations) for the four groups.

	Group					
	iRD	iSD	cRSD	TD	*F*	*p*
*N*	17	21	35	35		
Girls/boys	10/07	09/12	17/18	17/18		
Age (months)	123.71 (4.33)	125.71 (5.97)	123.8 (5.43)	123.29 (4.66)	1.04	.379
Non-verbal IQ ^1^	115.88 (13.26)	109.48 (13.11)	107.97 (12.30)	109.97 (10.87)	1.65	.182
ADHD score	0.37 (0.16) _b_	0.63 (0.31) _a_	0.51 (0.31)	0.42 (0.30)	3.21	.026
Reading (SLS) ^2^	10.48 (4.65) _bd_	40.13 (18.37) _acd_	9.65 (9.15) _bd_	52.23 (13.02) _abc_	86.32	< .001
Reading (SLRT-II)^2,3^	15.06 (3.47) _bd_	42.98 (12.90) _acd_	11.40 (6.30) _bd_	56.01 (15.83) _abc_	109.79	< .001
Spelling ^2,3^	42.72 (13.16) _bcd_	14.79 (6.22) _ad_	9.63 (6.02) _ad_	57.71 (10.99) _abc_	190.32	< .001
Digit span (fw) ^4^	7.82 (1.43)	7.52 (1.57)	7.43 (1.65)	7.54 (1.38)	0.26	.853
Digit span (bw) ^4^	6.71 (1.40) _c_	6.19 (1.40)	5.69 (0.90) _ad_	6.46 (1.09) _c_	3.97	.010
PA (% correct)	73.31 (15.70)	73.88 (16.65) _d_	65.18 (17.61) _d_	84.91 (8.74) _bc_	10.52	< .001
RAN (items/s)	1.89 (0.35) _bd_	2.30 (0.33) _ac_	1.87 (0.36) _bd_	2.38 (0.52) _ac_	11.80	< .001

1 IQ scores (*M* = 100; SD = 15).

2 Percentile rank.

3 Average scores of grades 3 and 4.

4 Raw scores (maximum score = 16).

PA: phonological awareness; RAN: rapid automatized naming.

Subscript letters indicate that the mean differs reliably (*p *< .05) from the referred-to mean (*post-hoc* Bonferroni tests): a = iRD (isolated reading deficits), b = iSD (isolated spelling deficits), c = cRSD (combined reading and spelling deficits), and d = TD (typically developing children). Due to extreme outliers, one control child was excluded from the analysis of RAN.

### Procedure

The reading screening test (SLS 2–9), the spelling test at the end of grade 3, and the non-verbal IQ test were administered in classroom settings, while all other measures were administered individually at the Medical Center of the University of Munich.

### Material

#### Reading

A standardized reading speed test (SLS 2–9; Wimmer & Mayringer, 2014; parallel-test reliability *r* = .95 and content validity *r* = .89 for grade 2) was given as classroom measure. Children were asked to read simple sentences silently and to mark them as semantically correct or incorrect (e.g., “Trees can speak.”). After 3 minutes, the task was terminated and the number of correctly marked sentences was scored.

In addition, an individually administered 1-minute reading fluency test (SLRT-II; Moll & Landerl, 2010; parallel-test reliability *r* = .90–.94 and content validity *r* = .69–.85 for grade 3) was administered in grades 3 and 4. The test contains a word and pseudoword reading list with items increasing in length and complexity. Children were asked to read each list aloud as fast as possible without making any errors. The relevant measure is the number of correctly read words and pseudowords within the 1-minute time limit.

#### Spelling

Spelling was assessed twice using standardized spelling tests. At the end of grade 3, the DRT-3 (Müller, 2003; parallel-test reliability *r* = .92 and content validity *r* = .78) was administered. The task consisted of 44 single words, which had to be written into sentence frames. The experimenter dictated the words. The number of correctly spelled words was scored. At the end of grade 4, spelling was assessed by the DERET 3–4+ (Stock & Schneider, 2008; parallel-test reliability *r* = .95 and content validity *r* = .79 for grade 4). Children were asked to write 10 sentences (92 words), which were dictated by the examiner. The number of misspelled words was scored.

#### Non-verbal IQ

The German version of the Culture Fair Intelligence Test (CFT 20-R; Weiss, 2006) was administered in order to estimate non-verbal cognitive abilities. The test consists of four subtests: Series, Classification, Matrices, and Topology and has a high reliability (*r* = .92-.96) and content validity (correlation with the “g”-factor *r* = .78-.83).

#### Attention rating

In order to exclude children with clinically significant ADHD symptoms and to assess attentional (sub-)clinical problems, we conducted a telephone interview with one of the participant’s caregivers based on the ADHD questionnaire of the DISYPS-II (Döpfner et al., 2008). The DYSIPS-II is a well-established standardized structured interview for psychiatric disorders based on DSM-IV (Saß &American Psychiatric Association, 1998) and ICD-10 (World Health Organization, 1992) guidelines (Cronbach’s alpha = .87–.94 for parental ratings of ADHD symptoms). The ADHD-questionnaire comprises 20 questions with a 4-point rating scale corresponding to the three main dimensions of ADHD symptoms: attentional deficits (nine items), hyperactivity (seven items) and impulsivity (four items). An ADHD score >1.55 for boys and 1.10 for girls indicates attentional clinical problems. Children scoring above this cutoff were excluded.

#### Verbal memory

Verbal short-term and working memory was assessed by the Digit Span forwards (fw) and backwards (bw) subtest of the German version of the Wechsler Intelligence Scale for Children (WISC-IV; Petermann & Petermann, 2011).

#### Phonological awareness

PA was assessed by a computerized phoneme deletion task using pseudowords which was developed in our lab. The task was programmed with Presentation 18.3 (Neurobehavioral Systems, Inc., Berkeley, CA, USA) and consisted of 4 practice and 25 test trials (20 mono- and 5 disyllabic pseudowords), which were presented via headphones. Children were asked to repeat each pseudoword first and then had to pronounce it without a specified phoneme (e.g., “/fɔlt/without/t/” – /fɔl/). The experimenter marked responses for correctness. Any pseudoword that was not pronounced correctly was replayed up to three times. The ratio of correct responses to the total number of responses was scored. Cronbach’s alpha was .78.

#### Rapid automatized naming

A standard RAN-digits paradigm (Denckla & Rudel, 1976) was presented. Children had to name a matrix of 40 monosyllabic digits (8, 3, 5, 2, 9) presented in eight lines and five columns as quickly and accurately as possible. Item order was randomized and each item was presented once in each line. Children were familiarized with the task with a 3 × 5 RAN array format. The time needed to name the full item set and any occurring errors were recorded and transformed into items named correctly per second.

### Orthographic learning task

#### Stimulus material

As described above, German orthography contains a high number of orthographic markers. Particularly relevant is the correct marking of vowel length. Differentiating between short and long vowels provides important information about the spelling of a word. For example, short vowels are always followed by at least two consonants. However, differentiating between short and long vowels is not sufficient to know the exact spelling of a word. This is especially the case for words with long vowels because the same long vowel can be spelled in different ways. For example, the long vowel/a:/can be spelled with the single letter *a* followed by one consonant (e.g. *Tal* – valley), with the single letter *a* followed by the silent letter *h* (e.g. *Wahl* – election), or with two letters *aa* (e.g. *Saal* – hall). Thus, in order to spell a word correctly children need to differentiate between short and long vowels, and also need to store the exact orthographic marker of the word in memory. Note that long and short vowels differ only in vowel duration, while differences in vowel quality are minimal (e.g.,/ʃa:l/- scarf vs./ʃal/- sound). In the current study orthographic markers associated with vowel length were used to assess whether orthographic learning had taken place. Therefore we constructed eight pronounceable monosyllabic pseudowords including these markers (Blotz, Mochs, Natz, Miel, Toof, Behn, Kohs, Naal). In addition to the eight target names we developed distractors which were used in the posttest reading task: For each target name we constructed (1) a pseudoword with the same vowel length (homophones), (2) a pseudoword with different vowel length but otherwise same pronunciation, and (3) a pseudoword that is visually similar to the target name, but has a different pronunciation (see Appendix A). Comparing the target with the different non-target stimuli provides information about children’s orthographic knowledge, sensitivity to vowel length, and visual discriminability during reading. We checked in the CELEX database for German (Baayen, Piepenbrock, & Rijn, 1993) if the pseudowords of these four conditions differed regarding bigram frequency (*F*(3, 28) = .36, MSE = 42,498,607.74, *p* = .78) and neighborhoods (*F*(3, 28) = .05, MSE = 5.80, *p* = .99) and found no significant differences. All stimuli contained only permissible letter patterns and had regular pronunciations.

To assess whether difficulties in orthographic learning are restricted to verbal material, we included a non-verbal control condition, with eight symbols (SPSS Marker Set, see Appendix B). Symbols were constructed to be visually simple but sufficiently complex in order to avoid that a symbol could be associated with a single verbal label (such as circle, triangle etc.). Therefore, each symbol comprised several visual features (e.g., “triangle with a filled circle in the middle”) which made it more difficult to name them. This was done to ensure that the control task tapped visual rather than verbal skills.

#### Procedure of the orthographic learning task

**Figure 1.**
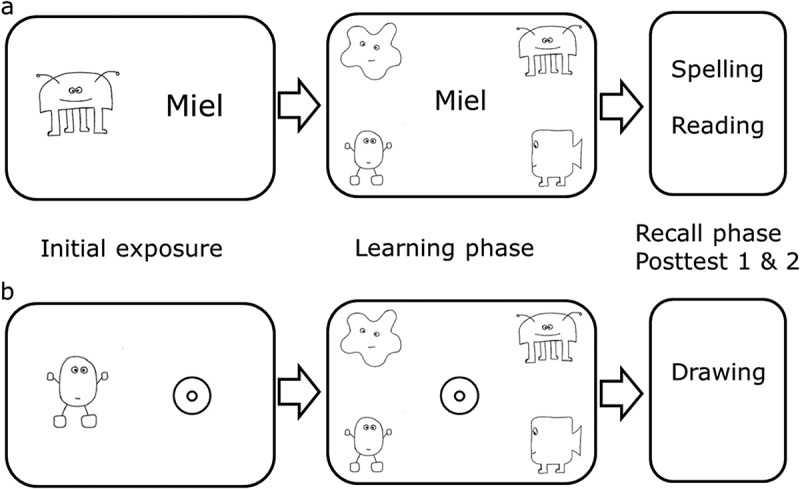
Procedure of the learning tasks (a = verbal learning task: nonsense names, b = non-verbal learning task: symbols).

Children were tested individually in a quiet room. Half of the children (randomly selected) initially learned the eight pseudowords (in two blocks of four pseudowords) followed by the eight symbols (also in two blocks of four), while the other half learned the symbols first followed by the pseudowords (names). The presentation order of the two name and two symbol blocks was counterbalanced across participants, resulting in four possible presentation orders. For both conditions (names/symbols), the same procedure was used: an initial exposure phase, a learning phase and a recall phase (including two posttests: immediate and delayed recall) (see Figure 1). Exposure phase and learning phase were computerized tasks programmed with E-Prime 2.0 software (Psychology Software Tools, Inc).

#### Initial exposure phase

Each learning task started with an initial exposure phase, which was similar to the exposure phase described by Messbauer and de Jong (2003). The child was seated in front of a computer screen and the experimenter showed a picture of one of eight alien creatures. Children were told that they were going to learn the names (symbols) of altogether eight aliens (presented in two blocks of four). Next, the experimenter presented each alien creature one after the other either together with the corresponding name (= verbal learning task), or with the corresponding symbol (= non-verbal learning task). In the verbal learning task, the experimenter named the name aloud and the child was asked to listen carefully, and to repeat the nonsense name pronounced by the experimenter in order to ensure that the child had perceived the name correctly and could pronounce it accurately. The child was asked to try to remember the nonsense name as well as the spelling of the name that was presented together with the alien creature on the screen. Thus, a connection between the spelling (orthography) and pronunciation (phonology) was provided. In the non-verbal learning task, the child was asked to watch carefully and to draw the symbol shown on the screen in order to ensure that the child could draw the symbol accurately. For both conditions feedback was given. The aim of the initial exposure phase was to familiarize the child with the task and to introduce the names and symbols. Each name/symbol was only presented once to the child. The initial exposure phase was neither scored nor included in the analyses.

#### Learning phase

The initial exposure phase was followed by a paired associate learning task. The child first had to read the nonsense name which was presented in the middle of the screen, and then had to match the name (or the symbol) by pointing at the correct alien creature out of four presented aliens. The experimenter scored the child’s response by pressing the corresponding key on the keyboard. Irrespective of the child’s response, the correct alien was highlighted as feedback on the screen and the experimenter always pronounced the nonsense name correctly. The assignment was scored as correct or incorrect. Five assignment trials for each name/symbol were given, thus the maximum score for each condition was 40 (8 names/8 symbols × 5 test trials). In the verbal condition, children are supposed to build-up orthographic representations of the spellings by repeatedly seeing and reading the alien names when assigning them. This assumption is based on Share’s self-teaching hypothesis (1995) describing that phonological processing and repeated decoding plays an important role in building-up word-specific orthographic representations.

#### Recall phase

The learning phase was followed by a recall phase including two posttests: Posttest 1 was carried out immediately after the learning phase (immediate recall) and posttest 2 was carried out 2 hours later (delayed recall). Between the two posttests the children were given other cognitive tasks as part of the larger project. At both posttests (immediate and delayed recall) orthographic learning in the name condition was measured first by a spelling and then by a reading task. This sequence was chosen because the reading recall task included not only the target words but also words with incorrect orthography and/or incorrect phonology. In order to avoid that children were exposed to false spellings or different words directly before assessing the spelling outcome, we administered the spelling task first. Symbol learning in the symbol condition was measured by a recall (drawing) task. No corrective feedback was given for any of the posttests.

In the *spelling task* (production of word-specific representations), the experimenter dictated each of the trained pseudowords (alien names) and the child was asked to write down the aliens’ names exactly as they had learned them, thus the child was asked to take into account the correct orthographic marker of the learned word. The maximum score was eight. For the children who made errors, we carried out an error analysis, which distinguished between two types of errors: orthographic errors that did not violate vowel length (e.g., Mil instead of Miel) and other kind of errors, either violating vowel length (e.g., Mill instead of Miel) or indicating phonetically incorrect spellings (e.g., Niel instead of Miel). A spelling-to-dictation task was used because producing a word is more difficult than recognizing the spelling of a word (e.g. in lexical decision tasks) and is therefore better suited to assess the quality of orthographic representations.

In the *reading task* (recognition of word-specific representations), the child was asked to read aloud as accurate and fast as possible a list of 32 pseudowords. The list included the eight previously learned target words (alien names), eight pseudowords of the same vowel length (homophones), eight pseudowords of different vowel length, and eight pseudowords of visual similarity (see Appendix B for the whole item set). Items were presented one after the other in a fixed randomized order on a computer screen; each item was preceded by an acoustic signal. Three practice items preceded the test items. The items appeared in the center of the screen, 32-point type, in yellow on black background, using Presentation 18.3 software. Reaction times (from onset of the stimulus to onset of the participant’s oral response) were measured by a voice key while reading was tape-recorded. When the voice key was triggered, the color of the presented item changed, allowing the experimenter to check whether the voice key was triggered correctly. Accuracy data were recorded by the experimenter. Interrater reliability indicated almost perfect agreement (κ = .90, *p *< .001). The experimenter marked the response to each item as “correct,” “incorrect,” or “check later” by pressing different response keys on the keyboard. “Check later” indicated that the voice onset of the child did not match the response latency measured by the voice key. This was the case when the voice of the child was too soft to trigger the voice key or when an earlier background noise triggered the voice key before the child responded. The next item was presented 1000 msec after the key press of the experimenter. The maximum accuracy score was 32 (4 conditions x 8 pseudowords). In addition to the voice key recordings, all items were measured on the basis of the tape-recordings using Audacity 2.1.2 software. To check interrater reliability we compared the measured voice onset times (VOTs) for all items of one participant in the immediate reading posttest and found high correlation between the experimenters (*r *= .996, *p *< .001). Depending on the quality of recordings, the tape recordings or the voice key response times were used for analysis. The VOTs of the two methods showed a very high correlation (*r *= .961, *p *< .001).

For the *symbol recall task*, the child received an A4 sheet of paper with the pictures of the eight aliens and was asked to draw the symbol associated with each creature. The maximum score was eight.

## Results

### Participant descriptives

Table 1 shows performance of the four groups of children on the selection literacy measures and on cognitive skills. Regarding age and non-verbal IQ the groups did not differ from each other. Regarding attention the iSD group showed a significantly higher score (indicating more ADHD-symptoms) compared to the iRD group. However, please note that none of the children scored above the cutoff indicating clinically relevant symptoms of ADHD. In line with the selection criteria, the groups differed in terms of their reading and spelling skills. Importantly, the two groups of dysfluent readers (iRD, cRSD) did not differ in their reading performance, and the two groups of poor spellers (iSD, cRSD) did not differ in their spelling performance. All three deficit groups (iRD, iSD, cRSD) showed lower performance than controls on the reading and spelling measures. While the four groups did not differ in verbal short-term memory (digit span forwards), children with combined reading and spelling problems (cRSD) showed a significantly lower performance in verbal working memory as measured by digit span backwards than the two groups with adequate spelling skills (iRD, TD). On PA the two groups of poor spellers (iSD and cRSD) performed significantly lower than controls. Regarding the automatized naming (RAN) measure, the fluent readers (iSD and TD) performed better on RAN than the two groups with reading fluency deficits (iRD and cRSD).

### Performance in the learning phase (paired associate learning)

Performance (accuracy) in the learning phase was not normally distributed. Therefore nonparametric analyzes (Kruskal–Wallis tests) were run separately for the two conditions: name learning and symbol learning. Table 2 shows the mean accuracy values of the four groups. There were no significant differences between the groups for both, name learning (*H*(3) = 3.67, *p* = .30) and symbol learning (*H*(3) = 1.19, *p* = .76).

**Table 2.** Mean number of correct matches during learning phase (SD in parentheses, max. score = 40, five trials of eight items).

	Group			
	iRD	iSD	cRSD	TD
Name learning	36.18 (3.24)	35.10 (4.71)	33.69 (4.54)	34.26 (5.44)
Symbol learning	36.41 (3.28)	35.57 (4.01)	34.60 (5.11)	35.19 (4.57)

### Recall phase: Spelling

A repeated-measures ANOVA was run with time (posttest 1, posttest 2) as within-subject factor and group (iRD, iSD, cRSD, TD) as between-subject factor. Significant results were followed by *post-hoc* LSD tests. A significant effect of time (*F*(3, 104) = 9.35, MSE = .671, *p* < .01) and group (*F*(3, 104) = 5.02, MSE = 2.99, *p* < .01) was found. As expected, children spelled more alien names correctly directly after the learning task (posttest 1) than 2 hours later (posttest 2). The group main effect reflected the significantly lower performance of the combined deficits group (cRSD) compared to the two groups of good spellers (iRD, TD). The iSD group scored in-between the two groups of good spellers and the combined deficit group and did not differ significantly from any of the other three groups (iRD, cRSD, TD). Importantly, the main effect of group was modified by a significant interaction between time and group (*F*(3, 104) = 4.22, MSE = .671, *p* < .01). Univariate statistics for each posttest separately were conducted and followed by *post-hoc* LSD tests. Both, for posttest 1 (*F*(3, 104) = 5.76, MSE = 1.33, *p* = .001) and posttest 2 (*F*(3, 104) = 4.37, MSE = 2.33, *p* < .01) significant group differences were found (see Table 3).

**Table 3.** Mean number of correct name spellings and correct symbol drawings during recall (SD in parentheses, max. score = 8).

		Group			
		iRD	iSD	cRSD	TD
Spelling recall	Posttest 1	6.88 (1.22) _c_	6.95 (0.92) _c_	5.89 (1.39) _abd_	6.80 (0.96) _c_
	Posttest 2	7.06 (1.39) _bc_	5.86 (1.91) _a_	5.63 (1.56) _ad_	6.54 (1.29) _c_
Symbol recall	Posttest 1	4.88 (1.50)	4.24 (2.12)	4.23 (1.63)	3.89 (1.86)
	Posttest 2	4.71 (1.96)	3.81 (2.71)	3.71 (2.05)	3.43 (2.29)

Subscript letters indicate that the mean differs reliably (*p *< .05) from the referred-to mean (*post-hoc* LSD tests): a = iRD, b = iSD, c = cRSD, and d = TD.

**Figure 2.**
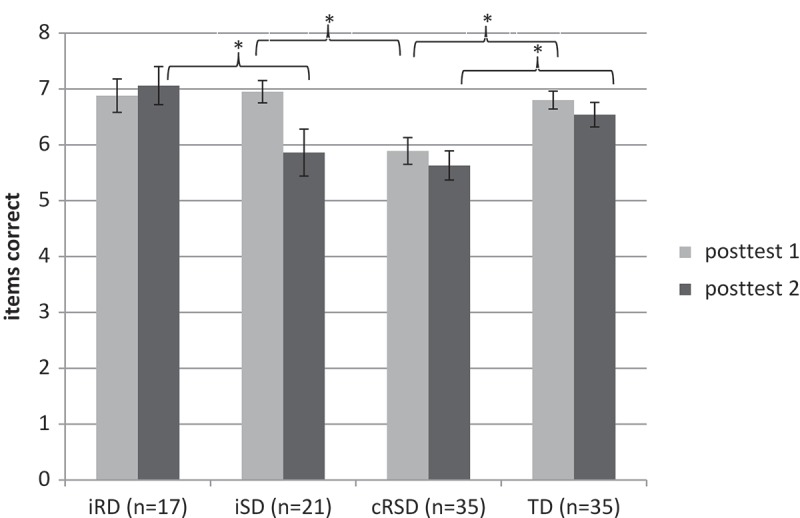
Mean scores for the spelling recall task for all four groups.

The analyses revealed that the interaction was due to the fact that directly after the associate learning task (immediate recall), children with iSD were able to spell most of the alien names correctly. They did not differ from the two groups of good spellers (*ps* > .05) and performed significantly better (*p* < .01) than the group with combined deficits (cRSD). However, 2 hours later in the delayed recall the performance of the iSD group decreased significantly (*t*(1, 20) = 3.65, *p* < .01) and was at the same level as the performance of the cRSD group. The other three groups showed no differences in their spelling accuracy performance between recall 1 and recall 2. Figure 2 illustrates this effect.

**Figure 3.**
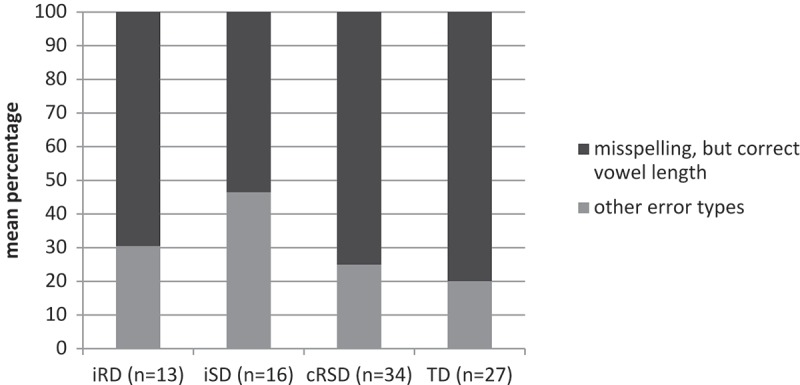
Mean percentages of the two types of errors for all four groups.

Next we compared the type of spelling errors in the four groups. Error types were not normally distributed. We therefore ran nonparametric analyzes (Kruskal–Wallis test) for the two kinds of errors: misspelling, but correct vowel length and other error types. Since only children who have made spelling errors are considered for this analysis, a new sample size was obtained: 13 children with iRD, 16 with iSD, 34 with cRSD and 27 TD. Although the absolute error rate was highest in children of the cRSD group (Figure 2), the relative distribution of the two error types was highly similar to the iRD and TD groups (see Figure 3). Results revealed again a different pattern for the iSD group compared to the other three groups. Although the main effect of group for each error type was only marginal significant (*χ*2 = 7.06; *p* = .07), results revealed that the iSD group seemed to have more problems in discriminating vowel length as they made more errors that violate vowel length and fewer misspellings with correct vowel length compared to the other groups. In addition, the iSD group showed a significant increase (*p* = .02) of errors that violate vowel length from immediate to delayed spelling recall. Thus, not only the number of correct spellings, but also the error pattern differed between the two groups of poor spellers.

### Recall phase: Reading

Table 4 summarizes the results of the reading task. Reading performance (accuracy and reaction time) was normally distributed. Repeated-measures ANOVAs were conducted with condition (target names, pseudowords of the same vowel length (homophones), pseudowords of different vowel length, visually similar pseudowords) as within-subject factor and group (iRD, iSD, cRSD, TD) as between-subject factor. Significant results were followed by *post-hoc* LSD tests.

**Table 4.** Means (standard deviations) for accuracy and reaction time (in sec.) for the reading recall task.

		Group			
		iRD	iSD	cRSD	TD
*N*		17	21	35	35
Acc.	Targets	7.00 (0.71) _c_	7.14 (0.92) _c_	6.41 (1.07) _abd_	7.39 (0.67) _c_
	Same vowel length	7.06 (0.73)	7.17 (0.62)	6.93 (0.81)	7.39 (0.71)
	Different vowel length	3.32 (2.06) _bc_	1.98 (1.21) _ad_	2.03 (1.06) _ad_	3.41 (1.66) _bc_
	Visual similarity	6.24 (1.06) _c_	5.64 (1.10) _d_	5.21 (1.11) _ad_	6.76 (0.87) _bc_
RT	Targets	1.30 (0.22) _bd_	1.08 (0.16) _ac_	1.25 (0.29) _bd_	1.07 (0.19) _ac_
	Same vowel length	1.41 (0.33) _bd_	1.14 (0.26) _ac_	1.30 (0.31) _bd_	1.15 (0.23) _ac_
	Different vowel length length	1.48 (0.53) _bd_	1.11 (0.21) _ac_	1.30 (0.34) _b_	1.17 (0.27) _a_
	Visual similarity	1.53 (0.41) _bd_	1.16 (0.22) _ac_	1.46 (0.47) _bd_	1.15 (0.27) _ac_

Subscript letters indicate that the mean differs reliably (*p *< .05) from the referred-to mean (*post-hoc* LSD tests): a = iRD, b = iSD, c = cRSD, and d = TD.

**Figure 4.**
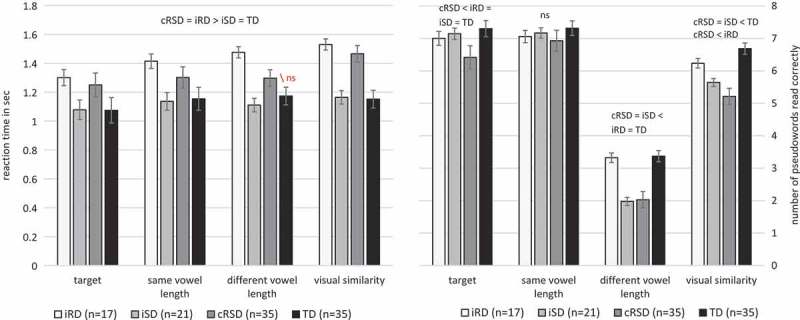
Mean scores for the reading recall task (left = reading reaction time, right = reading accuracy). Note: Error bars correspond to +/– one standard error; ns = not significant.

For *reading accuracy*, significant main effects of condition (*F*(3, 104) = 419.73, *MSE*  = .999, *p* < .001) and group (*F*(3, 104) = 19.26, MSE = 1.55, *p* < .001) were found. The condition effect indicated that it was particularly difficult to read items with different vowel length (see also Figure 4). Children often pronounced these pseudowords with the vowel length they had learned before. The group main effect reflected the significantly higher performance of the two groups of good spellers (iRD and TD) compared to the two groups of poor spellers (iSD and cRSD). In addition, the interaction between condition and group was significant, *F*(9, 104) = 3.09, MSE = .999, *p* < .01. Children with cRSD showed lower accuracy rates compared to the other three groups (all *p*s < .03) in reading target stimuli. For the condition “pseudowords of different vowel length” both groups of good spellers (iRD and TD) showed a significantly higher performance (*p *< .01) than poor spellers (iSD and cRSD). Regarding “pseudowords of visual similarity” the groups of poor spellers (iSD and cRSD) performed significantly worse (*p* ≤ .001) than controls (TD).

For *reaction time*, a significant effect of condition (*F*(3, 104) = 25.05, MSE = .015, *p* < .001) and group (*F*(3, 104) = 6.20, MSE = .332, *p* = .001) was found. The condition effect indicated that all groups were fastest in reading the target names and slowest in reading “pseudowords of visual similarity”. As expected and illustrated in Figure 4, the group main effect showed that the two groups of fluent readers (iSD and TD) read faster than the two groups of dysfluent readers (iRD and cRSD). The interaction between condition and group was also significant, *F*(9, 104) = 3.83, MSE = .015, *p* < .001. Posthoc tests revealed that fluent readers (iSD and TD) had shorter reaction times compared to dysfluent readers (iRD and cRSD) regarding all three conditions: target words (all *p*s < .01), “pseudowords of the same vowel length” (all *p*s < .04), and “pseudowords of visual similarity” (all *p*s < .01). In contrast, for “pseudowords of different vowel length” no significant difference was found between the cRSD group and the TD group (*p* = .13). Posthoc analysis within the groups revealed that all groups were faster in reading the target names compared to the “pseudowords with same vowel length” (*p*s < .03; iSD: *p* = .05), and the “pseudowords of visual similarity” (all *p*s < .01), but only the cRSD group was slower in reading “pseudowords of visual similarity” compared to “pseudowords of different vowel length” (*p* < .001). In order to include enough items in the reaction time analysis, both correctly and incorrectly read items were used. However, analysis only with correctly read items revealed the same significant main effects of condition and group.

### Recall phase: Symbols

For the symbol recall task, a repeated-measures ANOVA was run with time (posttest 1, posttest 2) as within-subject factor and group (iRD, iSD, cRSD, TD) as between-subject factor. Table 3 shows the descriptives. Only the main effect of time reached significance, *F*(3, 104) = 7.86, MSE = .967, *p* < .01, indicating that children remembered more symbols immediately after the learning phase (posttest 1) than 2 hours later (posttest 2). There was no significant difference between the groups (*F*(3, 104) = 1.35, MSE = 7.34, *p* = .26) and no interaction between time and group (*F*(3, 104) = .24, MSE = .967, *p* = .87). Thus, all children could equally well remember the symbols of the alien creatures.^2^2To examine whether the combined group reflects the sum of the deficits in the isolated groups, an ANOVA with a 2 (spelling deficit yes/no) × 2 (reading fluency deficit yes/no) design was carried out. In general, the cRSD group showed an additive profile characteristic of the isolated disorders for most dependent variables, including reading recall and delayed spelling recall. Only exception was an underadditive interaction found for immediate spelling recall.

## Discussion

The current study investigated orthographic learning in children with isolated reading fluency, isolated spelling and combined reading fluency and spelling deficits compared to controls.

First, we found no group differences on the paired associate learning task for both verbal and non-verbal material, suggesting that deficits in literacy skills are not associated with mapping of visual-orthographic information to visual-semantic information. The absence of difficulties in visual-visual paired associative learning in children with dyslexia has been reported in the literature before (Litt, de Jong, van Bergen, & Nation, 2013; Litt & Nation, 2014; Messbauer & de Jong, 2003). Thus, our findings extend previous research by showing that visual-visual paired associative learning is also unaffected in children with isolated literacy deficits. Previous studies could show that deficits in associative learning in children with dyslexia largely depend on the output modality and are only evident in tasks with verbal output, such as tasks where children had to remember and name a visually-presented symbol. In these visual-verbal tasks learning difficulties correlated with reading ability, indicating deficits in phonological form learning (Litt & Nation, 2014).

Second, we examined whether children differ in the spelling recall phase. In line with our hypothesis both groups of poor spellers were equally impaired in spelling recall at posttest 2. These findings are in line with previous studies showing that dyslexic readers make more errors than controls in spelling previously learned words during the recall phase (Bailey et al., 2004; Ehri & Saltmarsh, 1995; Share & Shalev, 2004; Wang et al., 2014).

However, a difference between the two spelling impaired groups (iSD and cRSD) emerged when analyzing performance in the spelling recall task at posttest 1, immediately after the learning phase. At posttest 1, only the cRSD group produced fewer spellings correctly than the control group, while the number of correct spellings in the iSD group did not differ from controls. Thus, for a short period of time children with iSD were able to produce the correct spellings, but 2 hours after the learning phase performance decreased to the level observed in the combined deficit group. These findings indicate that children with iSD seem to have specific problems in developing stable representations and storing these representations in long-term memory (=delayed recall). In contrast, children with cRSD already showed difficulties in building-up lexical representations. The analysis of the error types in spelling recall provided further information about the quality of these orthographic representations. Again, differences were found between the two groups of poor spellers. Children with iSD produced fewer misspellings with correct vowel length and made more errors that violate vowel length compared to children with cRSD. Furthermore, a significant increase of errors that violate vowel length was found in the iSD group only. Based on the lexical quality hypothesis (LQH) (Perfetti & Hart, 2002) high quality orthographic representations are both accurate and stable over time. Thus, the error pattern of children with iSD could reflect that they have unstable orthographic representations that allow them to spell a word accurately immediately after learning, but that are not precise enough to guarantee stability of correct spellings over time. When relying on unprecise representations orthographic markers are likely to be used incorrectly which often results in changes in vowel length. In contrast, children with cRSD showed problems in building-up orthographic representations (as evident from their poorer performance in the immediate recall task). Thus, children with cRSD might primarily rely on breaking down the dictated word in its constituting sound when spelling it, which results in phonologically correct spellings but without considering orthographic markers However, these interpretations are very speculative and need to be investigated in further studies that directly focus on error patterns.

Importantly, the difference in spelling performance between the two spelling impaired groups at posttest 1 (immediate recall) cannot be explained by the severity of the spelling deficit, given that the two spelling impaired groups did not differ in their initial spelling performance (selection criteria). A possible explanation for the difference between iSD and cRSD groups might be the poorer performance in verbal working memory (digit span backwards) in the cRSD group, which was not evident in the iSD group. Good verbal working memory skills in the iSD group might explain why children in this group were unaffected in immediate recall and only showed deficits when storing information in long-term memory. However, our data show that differences in working memory cannot solely explain the differences between the two groups of poor spellers, as the group difference remains significant after entering working memory as a covariate in the analysis. Although further research is needed to confirm this assumption, our interpretation with respect to the iSD group is in line with findings reported by Dreyer et al. (1995), who showed that poor spellers had less difficulties learning target spellings (immediate spelling test) than remembering the spellings one week later. However, the authors did not differentiate between poor spellers with and without reading problems. Another plausible explanation for the group difference in the spelling recall performance could be the influence of PA on orthographic learning. Interestingly, after entering PA as a covariate, the difference between cRSD and TD disappears in both recalls, while the difference between cRSD and iSD remains significant in the immediate recall and remains not significant in the delayed recall. Thus, it seems that PA has an influence on spelling performance in the cRSD group, but cannot explain the group difference of the two spelling impaired groups.

An important and novel finding is that children with iRD were not impaired in both spelling recall tasks (immediate and delayed recall), suggesting that their slow reading does not result from difficulties in learning, building-up and storing word-specific representations.

In line with our next hypothesis (2b), all children showed relatively high levels of reading accuracy across three of the four word conditions (targets, homophones, visual distractors). Although accuracy rates were generally high, the two groups of children with spelling problems showed significantly lower reading accuracy rates compared to the two groups of age-adequate spellers, particularly in the two non-homophonic conditions (“pseudowords of different vowel length” and “pseudowords of visual similarity”). These group differences support our assumption that children with spelling deficits have problems in building-up precise representations of previously learned words. Underspecified representations might be sufficient to recognize the targets correctly, but are likely to result in an increased error rate for non-homophonic pseudowords as a consequence of erroneously recognizing the distractors as target words. The result that children with combined reading and spelling deficits had problems in reading accuracy is in line with results of Reitsma (1983) and Ehri and Saltmarsh (1995), who found that dyslexic children had higher error rates compared to skilled readers. The finding that children with iRD showed no deficits in reading accuracy supports again the assumption that these children could build-up word-specific representations.

With regard to reading fluency and in line with our predictions (2c), children with reading fluency deficits (iRD and cRSD) read all stimuli significant slower than the two groups of fluent readers (iSD and TD). These findings were expected and reflect the selection criteria. However, the current findings can inform about why children with iRD read slower. Is it learning, storing or accessing of word-specific orthographic representations? As evident from their spelling skills in the standardized as well as in the experimental spelling recall tasks, children with iRD seem to have intact orthographic representations. Moreover, our findings showed that children with iRD also use their novel orthographic representations during reading, as they read the learned target words faster than the non-exposed pseudohomophone spellings. These findings suggest that reduced reading speed in the iRD group does not result from a deficit in building-up, storing and using orthographic representations, but is likely to result from a deficit in accessing or processing stored lexical representations. This interpretation is in line with other studies examining lexical access in German dyslexics (Bergmann & Wimmer, 2008; Gangl et al., 2017) and with findings showing a specific relation between rapid naming and overall reading speed for words as well as pseudowords (Moll, Fussenegger, Willburger, & Landerl, 2009; van den Boer, de Jong, & Haentjens-van Meeteren, 2013).

For children with isolated spelling problems predictions were less clear. As argued in the introduction, word-specific orthographic representations might not only be important for accurate reading and spelling but may also be relevant for fluent reading. Thus, it could be expected that children with iSD read slightly slower compared to controls, given that they have to rely on their incomplete orthographic representations and/or on compensatory decoding strategies. The fact that we did not find reduced reading speed in this group could be due to the fact that the experimental words were short and mono-syllabic. Slightly reduced reading speed in children with iSD might only become evident when items get longer and more complex or during passage reading. Support for this interpretation comes from the slightly poorer reading performance (although within the normal range) of children with iSD in the standardized reading tests (see Table 1).

Finally, we found no group differences in recalling symbols. This is in line with the few existing previous studies suggesting that there is no impairment in symbol learning in children with dyslexia (Messbauer & de Jong, 2003; Schulte-Körne et al., 2004). Thus, we extended these findings to children with isolated literacy problems by showing that they experience no problems in immediate and delayed recall of symbols. This indicates that children with spelling difficulties (iSD and cRSD) have no deficits in recalling non-verbal visual material but rather have specific problems in memorizing letter sequences of words (iSD) or in building-up word-specific representations (cRSD).

### Limitations and future research

First, the study focused on vowel length as one of the most relevant orthographic characteristics in German orthography. Future studies could examine vowel length more closely by looking at differences between items with short vowels (only one possible spelling) and items with long vowels (two possible spelling alternatives). Furthermore, there is need to clarify whether the current results generalize to other orthographical aspects.

Second, the present study focused on orthographic learning only and did not examine the influence of phonological memory. Given that we dictated the target words in the posttests, we do not know whether children were able to remember the pseudoword pronunciations. Results from previous studies with visual-verbal paired-associate learning paradigms (Litt et al., 2013; Mayringer & Wimmer, 2000) indicated that in tasks with verbal output, pseudoword learning correlated with reading ability and that children with dyslexia had difficulties in learning new phonological forms. Since these studies only examined children with cRSD (dyslexia), further research including children with isolated deficits are required.

Additionally, in the present study all participants, especially the TD showed relatively high accuracy rates both on associate learning and on spelling. In order to increase variance, future studies could include 10 stimuli and could further assess the number of exposures needed to reach a certain accuracy level.

Another aspect which should be taken into account in future research relates to the difference regarding the verbal and non-verbal condition in the initial exposure phase. In the verbal condition the children had to repeat the pseudoword while in the non-verbal condition the children had to draw the symbol. To make the procedure in both conditions more comparable, children could be asked to write down the pseudowords in the initial exposure phase.

Finally, future studies need to investigate how far the current findings can be transferred to less consistent orthographies, such as English. For example, for children with iSD differences in decoding depending on orthography have been reported. While English children with iSD show deficits in decoding (i.e., poor pseudoword reading) (Frith, 1985), decoding skills in children with iSD in consistent orthographies seem to be unaffected (see also pseudoword reading in the current sample). How these differences affect orthographic learning needs to be examined in future cross-linguistic research.

## Conclusions

The current findings support the assumption that different orthographic learning deficits underlie reading vs. spelling problems. Children with isolated reading (fluency) deficits seem to have no problems in building-up and storing word-specific orthographic representations in long-term memory since they showed no difficulties in both posttest spelling tasks. Furthermore, they did not differ in reading accuracy performance from control children, which also suggests that orthographic representations are intact. However, in the posttest reading tasks, children with iRD showed slower reaction times, suggesting that access to these intact representations or lexical processing is slowed down. For children with spelling problems we found differences between the two spelling impaired groups. Children with iSD showed no problems in immediate recall, but deficits in storing precise orthographic representations in long-term memory. In contrast, children with cRSD showed problems in building-up word-specific orthographic representations.

The error analysis further revealed that children with iSD seem to have specific problems in discriminating vowel length as they made more errors that violate vowel length compared to children with cRSD. This error pattern could reflect that children with iSD use unstable orthographic representations (Perfetti & Hart, 2002), while children with cRSD might primarily rely on decoding. These are important and novel findings which need to be examined in future studies.

## Highlights

- Orthographic learning was assessed in children with reading vs. spelling deficits.
- Reading and spelling problems were associated with different cognitive profiles.
- Reading problems were related to slow access to intact orthographic representations.
- Spelling problems were related to deficits in storing orthographic representations.
- No group differences were found for learning and recalling non-verbal material.

## Appendix A. Item set for the naming task with the corresponding phonetics

**Table:**

	Target pseudoword		Pseudoword with same vowel length (homophone)^a^	Pseudoword with different vowel length		Pseudoword with visual similarity	
	Miel	/mi:l/	Mihl	Mill	/mıl/	Miek	/mi:k/
	Kohs	/ko:s/	Koos	Koss	/kɔs/	Kohn	/ko:n/
	Naal	/na:l/	Nahl	Nall	/nal/	Naak	/na:k/
	Toof	/to:f/	Toov	Toff	/tɔf/	Toob	/to:b/
	Natz	/nats/	Nazz	Nahz	/na:ts/	Natt	/nat/
	Behn	/be:n/	Been	Benn	/bɛn/	Behs	/be:s/
	Mochs	/moks/	Mocks	Mooks	/mo:ks/	Mosch	/mɔʃ/
	Blotz	/blɔts/	Plotz	Blooz	/blo:ts/	Bloch	/blɔχ/
Bigram frequency^b^	7060 (4789)		5550 (6553)	6268 (6733)		8780 (7666)	
Neighborhood^b^	2.50 (1.60)		2.50 (1.77)	2.88 (2.42)		2.75 (3.41)	

a Phonetics are like targets. ^b^Means (and standard deviations).
